# Of numbers and movement – understanding transcription factor pathogenesis by advanced microscopy

**DOI:** 10.1242/dmm.046516

**Published:** 2020-12-29

**Authors:** Julia M. T. Auer, Jack J. Stoddart, Ioannis Christodoulou, Ana Lima, Kassiani Skouloudaki, Hildegard N. Hall, Vladana Vukojević, Dimitrios K. Papadopoulos

**Affiliations:** 1 MRC Human Genetics Unit, University of Edinburgh, Edinburgh EH4 1XU, UK; 2 Center for Molecular Medicine (CMM), Department of Clinical Neuroscience, Karolinska Institutet, 17176 Stockholm, Sweden

**Keywords:** Haploinsufficiency, Quantitative microscopy, Transcription factors, Super-resolution microscopy, Transcriptional regulation

## Abstract

Transcription factors (TFs) are life-sustaining and, therefore, the subject of intensive research. By regulating gene expression, TFs control a plethora of developmental and physiological processes, and their abnormal function commonly leads to various developmental defects and diseases in humans. Normal TF function often depends on gene dosage, which can be altered by copy-number variation or loss-of-function mutations. This explains why TF haploinsufficiency (HI) can lead to disease. Since aberrant TF numbers frequently result in pathogenic abnormalities of gene expression, quantitative analyses of TFs are a priority in the field. *In vitro* single-molecule methodologies have significantly aided the identification of links between TF gene dosage and transcriptional outcomes. Additionally, advances in quantitative microscopy have contributed mechanistic insights into normal and aberrant TF function. However, to understand TF biology, TF-chromatin interactions must be characterised *in vivo*, in a tissue-specific manner and in the context of both normal and altered TF numbers. Here, we summarise the advanced microscopy methodologies most frequently used to link TF abundance to function and dissect the molecular mechanisms underlying TF HIs. Increased application of advanced single-molecule and super-resolution microscopy modalities will improve our understanding of how TF HIs drive disease.

## Abstract (precis)

**Summary:** Abundance and chromatin interactions determine transcription factor function; their disruption can impair development and cause disease. We survey advanced microscopy methodologies that measure these properties and discuss molecular mechanisms that underlie transcription factor haploinsufficiency.

## Introduction

Precise tissue- and cell-specific regulation of gene expression is required for development and homeostasis. Transcription factors (TFs) tightly control transcriptional programmes in a cell- and tissue-dependent manner. General TFs and the core transcriptional machinery bind to gene promoters, whereas specific activating and repressing TFs bind to gene regulatory elements – i.e. transcriptional enhancers or silencers – and interact with promoter-bound complexes to control transcription (Horikoshi et al., 1988). This interaction is thought to occur through the Mediator complex, a multi-subunit protein complex that recognises enhancer-bound TF complexes and signals to RNA polymerase II to transcribe genes. The Mediator complex was first discovered in yeast (Kim et al., 1994); in humans, the basic Mediator complexes generally enhance transcription (Andrey et al., 2013; Bergiers et al., 2018; Ernst et al., 2011; Golan-Lagziel et al., 2018; Heintzman et al., 2009; Noordermeer et al., 2011; van Bömmel et al., 2018). Thus, cell type-specific gene expression is mainly influenced by the ability of TFs to bind their target sites within promoters or enhancers, which, in turn, relies on the chromatin state of these sites (Ernst et al., 2011).

The main TF families are classified according to their DNA-binding domains. These include the helix-turn-helix (HTH), zinc-finger (ZNF), basic helix-loop-helix (bHLH), basic leucine zipper (bZIP) and nuclear hormone receptor binding domains (Table 1), each featuring distinct mechanisms of sequence-specific DNA recognition and binding (Badis et al., 2009; Lambert et al., 2018; Wei et al., 2010). Furthermore, TFs often work in unique combinations, for example during development and differentiation (Bergiers et al., 2018). Thus, different cell types can be distinguished based on their TF repertoires. Thereby, the clustering of TF-binding sites in enhancer sequences favours functional synergies of TFs that are co-expressed in the same tissue (Golan-Lagziel et al., 2018).

**Table 1.**
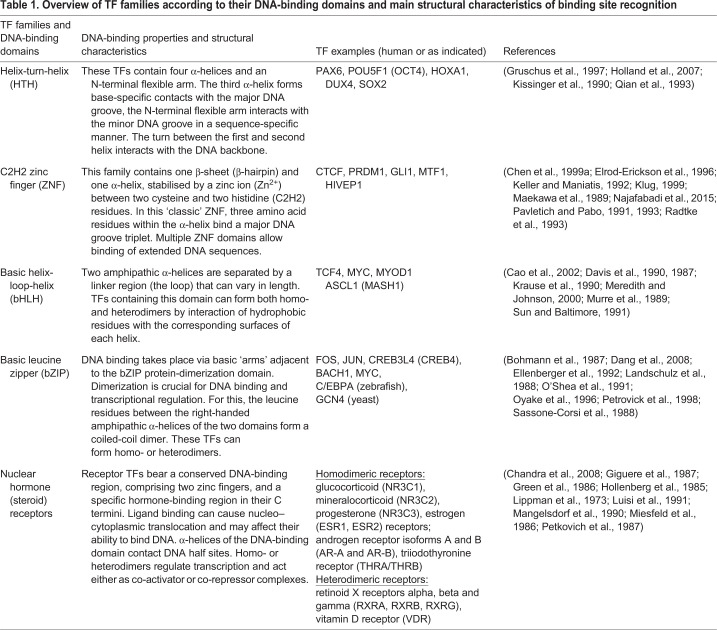
**Overview of** **TF families according to their DNA-binding domains and main structural characteristics of binding site recognition**

The aberrant function of TFs can have profound effects on development and disease. Of particular interest is how reduced levels of some TFs may influence their function and result in haploinsufficient phenotypes. Haploinsufficiency (HI) is the inability of a gene to rely on only one (of its two) alleles to exhibit its normal function. Therefore, HIs generally result in loss-of-function (LOF), often abnormal, phenotypes. It follows that HIs are properties of genes and, although ‘dosage-sensitive genes’ is a broader term than ‘HI genes’, henceforth, for simplicity, we use the two terms interchangeably.

LOF mutations or deletions of HI genes are inherited in a dominant manner, resulting in phenotypic alterations or abnormalities caused by the insufficiency of one intact allele to confer full gene functionality. Computational models aim to predict the tolerance of a system at the cell, tissue or organismal level to a single functional copy of a gene. Such models have mainly focussed on predicting the probability of genes being LOF-intolerant (pLI) (Lek et al., 2016), i.e. rating genes with a score of *n*≥0.9 as intolerant and with a score of *n*≤0.1 as tolerant to LOF mutations (Lek et al., 2016). Bioinformatics approaches and machine learning have identified 7841 HI genes in the human genome (Shihab et al., 2017), linking them to a number of dominantly inherited HI diseases. Key examples are eye disorders, such as aniridia (Jordan et al., 1992), keratitis (Mirzayans et al., 1995) and ocular colobomas (Sanyanusin et al., 1995; Williamson et al., 2014), as well as multiple cranial, facial and limb diseases – including synpolydactyly (Muragaki et al., 1996), schizencephaly (Brunelli et al., 1996), craniosynostosis of Adelaide type (Hollway et al., 1995; Jabs et al., 1993) and Greig cephalopolysyndactyly syndrome (Hui and Joyner, 1993; reviewed by Dang et al., 2008). Disease phenotypes have been attributed to ∼300 HI genes and range from primary immunodeficiencies (Franco-Jarava et al., 2018; Torgerson and Ochs, 2014) to ribosomopathies (Kyritsis et al., 2019) and cancer (Inoue and Fry, 2017; Jennings et al., 2012; Largaespada, 2001). Interestingly, a significant proportion of dosage-sensitive genes encode TFs, with higher relative numbers of these TF genes exhibiting HI compared to other human genes (Ni et al., 2019).

Over 1600 human TF genes have been grouped into gene families (Lambert et al., 2018), allowing for in-depth analysis of HI incidence in specific subsets of TFs (Table 1). Overall, 122 TFs from 32 gene families were identified and designated as the most reliable dosage-sensitive (MRDS) genes (Ni et al., 2019). This categorisation of TF families has attempted to explain the sensitivity – or insensitivity – of different TFs to dosage and found HI to be predominantly correlated with small TF families. Such small TF families – comprising fewer than eleven members, e.g. the Grainyhead and C2H2-ZNF/homeodomain families – are present in the MRDS dataset, whereas the largest subgroup of the zinc-finger TF family Krüppel-associated box (KRAB) domain-containing zinc finger proteins (KZFPs), is found to be dosage-insensitive and is not present in the MRDS dataset (Ni et al., 2019). This observation is yet to be explained, with evolutionary pressure being a plausible cause for limiting TF family size (Ni et al., 2019). Why other evolutionary mechanisms, such as gene duplication and divergence (Gehring et al., 2009), have not been deployed to expand these small families of TFs and alleviate HI remains unknown. Additional mechanisms that could account for such bias may relate to the regulatory, activating or repressing, behaviour of such TFs, or to the numbers of downstream-regulated genes. Thus, more work is needed to identify possible structural or functional similarities between dosage-sensitive TFs to further pinpoint the underlying disease-causing mechanisms of HIs. This Review summarises how the advanced quantitative microscopy methodologies aid in the study of biophysical and functional properties of TFs, such that TF dosage-sensitivity at the molecular level may be linked to development and disease.

## Factors resulting in HIs

**Fig. 1.**
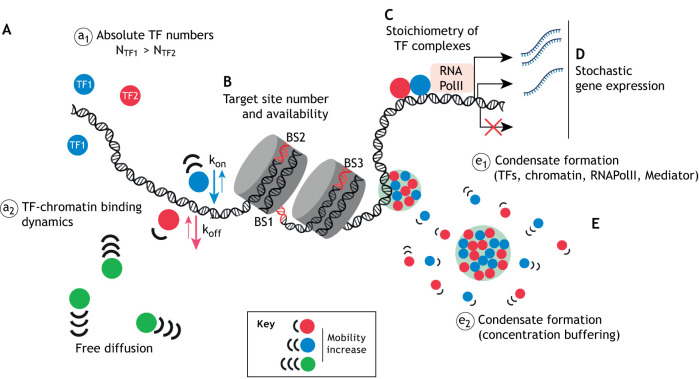
**Quantitative mechanisms that affect TF function.** (A) a_1_: N, absolute numbers of TF molecules. a_2_: Quantitative TF-chromatin binding dynamics. (B) Number and availability of TF-binding sites on chromatin (red DNA stretches represent TF-binding sites). (C) Stoichiometry of TF-interacting proteins. (D) Stochastic gene expression. (E) e_1_: TF co-condensate formation with chromatin, Mediator and RNA Pol II. e_2_: Intranuclear buffering of TF concentration caused by formation condensate. BS, binding site. Blue, red and green circles represent different TFs; grey cylinders represent nucleosomes; circles with a light green background represent condensates.
- stoichiometry of TFs and their interacting proteins;
- availability and binding kinetics to their TF target sites;
- stochastic nature of gene transcription;
- biophysical properties, such as their ability to form condensates.

Insufficient TF protein levels are the common denominator of haploinsufficient TF genes. In these cases, the abnormal phenotypes are triggered in a dosage-dependent manner (Gidekel et al., 2003; Gilchrist and Nijhout, 2001; Nishimura et al., 2001; Takeuchi et al., 2011). At the molecular level, altered TF numbers impair their functionality by interfering with their dynamic behaviour in cells and, thereby altering their transcriptional programmes (Bottani and Veitia, 2017; Veitia, 2003). We examine the following molecular and functional properties of TFs, which may underlie TF HIs in a tissue-specific manner (for a schematic representation, see Fig. 1):

- absolute TF numbers;

How these parameters vary simultaneously between or within cells, tissues or entire organisms adds to the sheer complexity in characterising the causes of dosage sensitivity and HI in humans (Elowitz et al., 2002; Veitia, 2002). HI may manifest in a range of related phenotypes in humans. Moreover, it is gradually becoming clear that not one single molecular mechanism is responsible for TF HIs; rather, parameters that determine TF function or dysfunction act in concert.

**Fig. 2.**
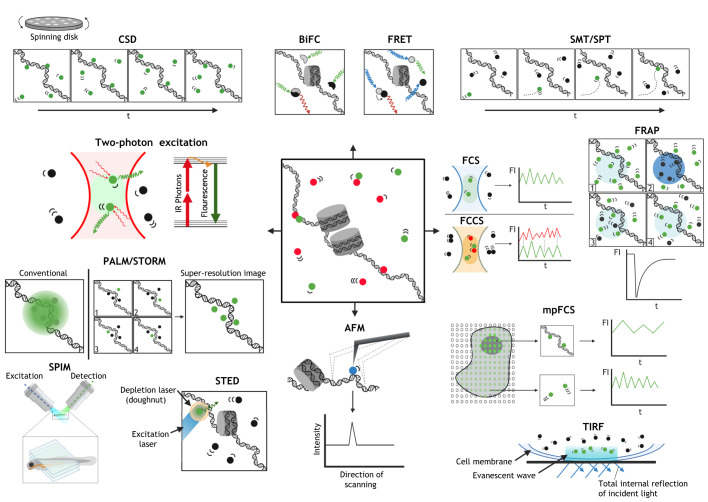
**Outline of quantitative microscopy methodologies used to study the concentration, dynamic behaviour, stoichiometry and subnuclear localisation of TFs.** For details on each approach, see Table 2.

Studying the mechanisms of TF concentration and function in TF HIs is becoming increasingly possible thanks to the continuous advancement of microscopy methods. Table 2 briefly outlines these methodologies and their potential applications to investigate TF biology, and selected methodologies are depicted in Fig. 2.

**Table 2.**
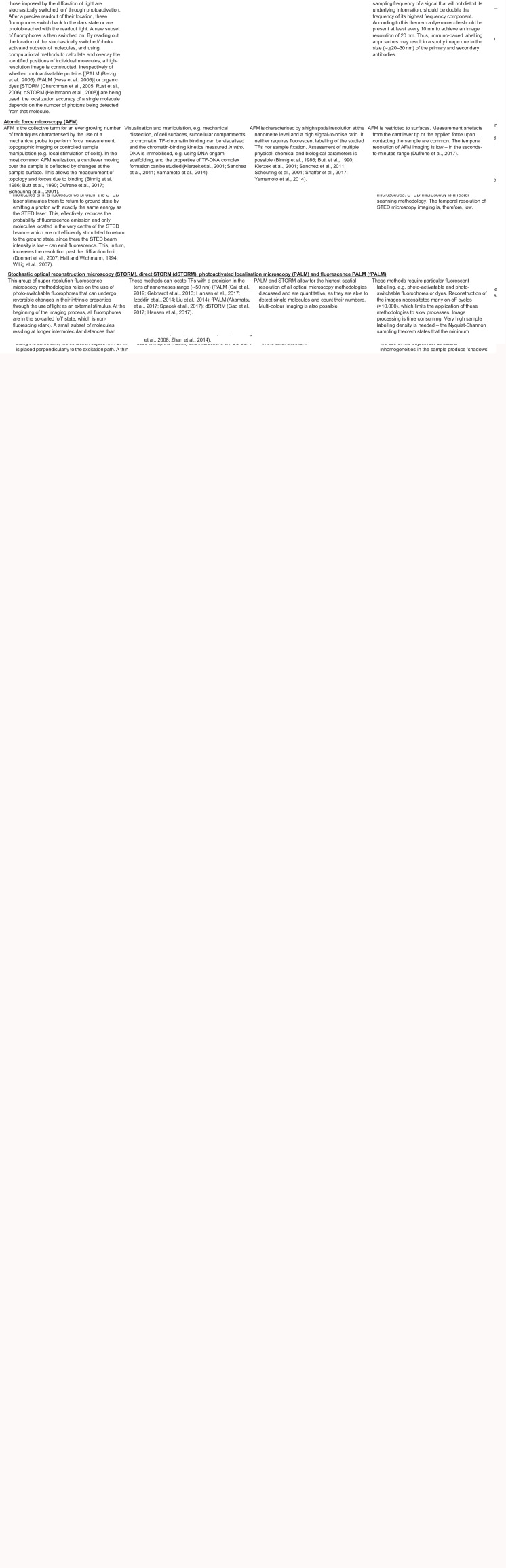
**Overview of the main advanced microscopy methodologies used to study TF dynamic behaviour, concentration, stoichiometry and localisation**

## Absolute TF numbers

Most TFs are expressed and, typically, act in a cell type- or tissue-specific manner. RNA sequencing (RNA-seq) analyses of human TFs found over a third of them to be enriched in specific tissues (Lambert et al., 2018; Uhlen et al., 2015). Spatiotemporally precise TF levels are key to driving cell specification and differentiation. Particularly during early development, different levels of stemness TFs control the fate of individual cells. For example, in mouse blastocysts, the distribution of SOX2 and POU5F1 (hereafter, referred to as OCT4), two of the key stemness TFs, confers an initial differentiation bias at the 4-cell stage (Goolam et al., 2016; Kaur et al., 2013; White et al., 2016). Later in embryonic development, differential expression of *NANOG* and *GATA6* in a ‘salt-and-pepper’ pattern in the mouse embryonic inner cell mass controls the segregation of the epiblast from primitive endoderm lineages (Chazaud et al., 2006). Stemness TF levels continue to control the pluripotency of different stem cell populations in development (Adachi et al., 2013). For instance, the intrinsically fluctuating amounts of OCT4 and SOX2 contribute to the lineage commitment of mouse embryonic stem cells (ESCs) during differentiation (Strebinger et al., 2019). When *Pou5f1* and *Sox2* become downregulated, mouse ESCs can differentiate and activate the trophoblast stem cell TF networks required for normal embryonic development (Adachi et al., 2013; Masui et al., 2007; Niwa et al., 2000, 2005). This continued dependence on concentration for the precise function of TFs during development explains why aberrant TF gene dosage can have detrimental effects on development and physiology. Changes to the required concentration of TFs at different points in development can elicit diverse effects in gene regulation and, thus, result in different pathogenic phenotypes.

At the subnuclear level, local concentrations of TFs affect gene expression by controlling the kinetics of TF binding to chromatin, recruitment of RNAPolII and transcriptional bursting (Nelson et al., 2004; Senecal et al., 2014). In mouse ESCs, the clustering of SOX2-bound enhancers modulates the search for local SOX2 target sites and facilitates gene transcription (Liu et al., 2014). RNAPolII activity at the *POU5F1* enhancer is regulated by the accumulation of SOX2 and the chromatin reader BRD4, their chromatin-binding dynamics, as well as their interactions with the Mediator complex and the elongation controller P-TEFb, a cyclin-dependent kinase consisting of CDK9 and one of several cyclin subunits (Li et al., 2019). The subnuclear localisation of TFs may also vary between cell cycle stages and many TFs (including SOX2) become enriched on mitotic chromosomes, a phenomenon referred to as ‘mitotic bookmarking’ (Caravaca et al., 2013; Dufourt et al., 2018; Kadauke et al., 2012; Young et al., 2007). This local enrichment is mediated by the active nuclear import of TFs (Teves et al., 2016). TF binding persists through cell divisions, thus conferring transcriptional ‘memory’. At the cellular level, the concentration of TFs may provide cells with a molecular ‘readout’ of their relative positions in a tissue. For example, in *Drosophila* embryos, the chromatin-binding activity of Bicoid (Bcd), a maternally provided TF and key morphogen, which determines embryonic anteroposterior polarity, remains unaltered despite its low levels within posterior nuclei. This is achieved when Bcd binds hubs together with the pioneer TF Zelda (Zld), which results in increased local concentrations (Mir et al., 2017). Thus, while distinct concentrations of TFs may elicit differential cell fates, cells can deploy alternative mechanisms to preferentially counteract the effect of different TF concentrations when needed.

Although TF function has been extensively studied in flies, the latter are a rather inappropriate model to study TF HIs, as the majority of heterozygous mutations do not cause observable developmental defects. This may be explained by the fact that the *Drosophila* genome is ∼20 times smaller than the human genome, but the average concentration of TFs regulating this smaller genome is not proportionately decreased. Additionally, extensive endoreplication, which takes place in several fly tissues, produces polyploid cells during development, which are expected to be able to better buffer low TF gene dosage than diploid cells (Sher et al., 2013). However, gene regulation by TFs has been studied very extensively in this organism, therefore, we discuss crucial findings from *Drosophila*. Taken together, the discussed findings support the notion that the absolute number of TFs is an important determinant affecting gene expression by means of various mechanisms.

The importance of TF numbers and their subnuclear localisation for normal function are an enticing subject of research. The absolute number of TFs can be measured in living or fixed cells by fluorescence correlation spectroscopy (FCS) or super-resolution microscopy (SRM), respectively (Table 2 and Fig. 2). FCS allows the measurement of TF numbers and their molecular mobility with high temporal resolution (Ehrenberg and Rigler, 1974; Papadopoulos et al., 2010; Vukojevic et al., 2010). Additionally, the spatial variability of TF numbers within or between cell nuclei can be simultaneously analysed by employing massively parallel FCS (mpFCS) that uses an array of detectors coupled to diffractive optical elements (EM-CCDs) (Capoulade et al., 2011; Krmpot et al., 2019; Papadopoulos et al., 2015), or by selective plane illumination microscopy (SPIM) coupled to fast cameras, such as electron-multiplying charge-coupled devices (EM-CCDs) (Capoulade et al., 2011; Krieger et al., 2015, 2014; Wohland et al., 2010). FCS has been used to study the dynamic behaviour of TF binding to chromatin, such as in the case of the formation of the Bcd gradient during early *Drosophila* development (Abu-Arish et al., 2010), the TF-chromatin binding dynamics of SOX2-OCT4 (Chen et al., 2014b), TetR (Normanno et al., 2015), the specific and non-specific binding of the Hox TF Sex combs reduced (Scr) (Papadopoulos et al., 2010; Vukojevic et al., 2010) and the variable dynamic binding behaviour of the TF MYC (Rosales et al., 2013). FCS has also been employed to study the variability in TF concentration and how this leads to the acquisition of differential developmental fates as, for example, in the case of the TF Senseless in the *Drosophila* wing imaginal discs (Giri et al., 2020), FOS and JUN in HeLa cells (Szalóki et al., 2015) or the transcriptional co-activator Yorkie (the ortholog of the human YAP transcriptional co-activator) in different subcellular compartments of the developing *Drosophila* airways (Skouloudaki et al., 2019). Taken together, these studies show how diverse FCS methodologies can provide information on TF dynamic behaviour in live cells.

SRM methodologies, however, provide higher spatial resolution to visualise the distribution of subnuclear TFs and, therefore, complement FCS. Despite lacking the temporal and quantitative information, which FCS allows to derive by studying live samples, SRM methodologies provide excellent spatial information, which FCS methodologies cannot. Single-molecule spatial resolution may be achieved by limiting the number of fluorescent light-emitting molecules through selective illumination and/or sparse excitation (see Table 2 and Fig. 2). In these cases, TFs can be located − with high precision – on chromatin, whereas the concentration of TFs can still be inferred semi-quantitatively, by comparing their fluorescence intensity levels with those derived from a sample of known TF concentration (Lasker et al., 2020; Li et al., 2019; Reisser et al., 2018; Wollman and Leake, 2015). Furthermore, the quantification of TF transcripts can provide information about relative gene expression levels and, in some cases, may be used as a proxy to quantify TF abundance in a population of cells. Such relative measures of TF messages, their target transcriptomes and/or cell-to-cell heterogeneities of transcript numbers can be obtained by detection of RNA in single-cells. These measures scale with, albeit do not necessarily predict, TF variability at protein level and include single-molecule fluorescence *in situ* hybridisation (smFISH) and single-cell RNA sequencing (scRNA-seq). smFISH has been used to measure levels of *FOXO* mRNA (Blice-Baum et al., 2019), *NKX2-2* mRNA distribution in mouse pancreatic islet cells (Cui et al., 2018) and *OCT4*/*SOX1*/*T-BRACHYURY* transcription in differentiating mouse ESCs (Lanctôt, 2015). A main advantage of scRNA-seq is that it can distinguish between cells on the basis of their expressed mRNA repertoire (transcriptome profiling), and can measure the levels of individual TF messages, such as of *OCT4*/*SOX2* in developing mouse embryos (Goolam et al., 2016), or *PAX6* in a subclass of mouse cortical cells (Zeisel et al., 2015). The combination of microscopy-based and transcriptomics modalities allows researchers to precisely quantify the cell-to-cell variability of TF concentration at the protein or mRNA level, as well as to assess how such variability affects their target transcriptome. Overall, one can link the absolute amount of TFs to their function in transcriptional regulation. To understand how aberrant levels of TFs may lead to HI phenotypes, the absolute number of TFs needs to be efficiently measured in single cells and between different cells in a tissue. Therefore, quantitative methodologies need continuous advancement to become routinely accessible and accommodate a continuously increasing demand for precision.

## Stoichiometry of TF complexes

TFs rarely act alone. They predominantly bind target enhancers jointly with other TFs or cofactors to regulate the transcription of their target genes (Golan-Lagziel et al., 2018; Veitia, 2002). Hence, the formation of TF complexes depends on TF numbers, a phenomenon that appears to be conserved in evolution (Papp et al., 2003; Sopko et al., 2006; Veitia, 2002, 2003). A study by Ni et al. showed that dosage-sensitive TFs – which often drive HI phenotypes – interact with a total of 851 proteins, 25% of which are other TFs (Ni et al., 2019). Dosage-insensitive TFs, by contrast, interact only with 263 proteins, of which 17% are other TFs (Ni et al., 2019). Whether such TF-TF interactions are required for TF function and whether HIs of some TF genes are partially caused by abnormal TF stoichiometry within complexes remain to be shown. However, this disparity is striking and might shed light into the widespread downstream transcriptional effects triggered by reduced levels of individual TFs in HIs.

**Fig. 3.**
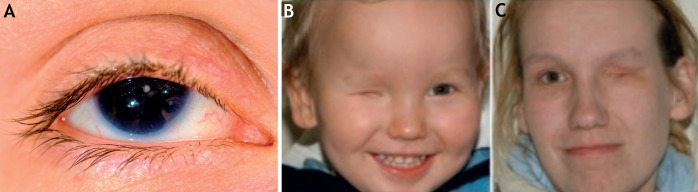
**Abnormal TF numbers and TF HI have important phenotypic consequences.** Human ocular developmental abnormalities can be caused by heterozygous mutations in the *PAX6* and *SOX2* TF genes. (A) Eye of a patient with a phenotype typical of *PAX6* HI, showing near-complete aniridia (absence of the iris) and mild ptosis (drooping of the eyelid). The patient had cataract surgery in young adulthood (Hall et al., 2019). Image courtesy of David Hall, Critical Care, Royal Infirmary of Edinburgh, UK. (B,C) An affected son (B) and his mother (C), both bearing a *SOX2* mutation that causes HI, which results in unilateral anophthalmia (absence of the eye). Although they carry the same mutation, their phenotypic abnormalities only affect the right or the left eye, respectively. Image adapted with permission from Gerth-Kahlert et al. (2013) under the terms of the CC-BY 3.0 license.

Many TF complexes display cooperative binding to chromatin, rendering the maintenance of stable cell type-specific transcriptional programmes reasonably sensitive to TF numbers (Bartman et al., 2016; Fukaya et al., 2016). This might explain why either a reduction or increase in individual TF protein levels can have deleterious effects on normal cellular function (Chen et al., 2008; Cox et al., 2010; Gao et al., 2012; Papp et al., 2003; Veitia, 2002; Wang et al., 2006; Wuebben et al., 2012). An extensively studied example of this is regulation of the ESC state by distinct TF combinations and levels (Chen et al., 2008; Cox et al., 2010; Gao et al., 2012; Wuebben et al., 2012). A second example is the cooperative binding between PAX6 and SOX2 during optic field induction. Here, both *Pax6* overexpression (e.g. through a third-copy allele) and heterozygous pathogenic mutations result in ocular developmental defects that are similar between mice (Ouyang et al., 2006; Schedl et al., 1996) and humans (Gerth-Kahlert et al., 2013; Hall et al., 2019) – see Fig. 3 for developmental abnormalities in the eye, caused by heterozygous *PAX6* and *SOX2* mutations. Such strong dependence on TF abundance, in which either increased or decreased TF numbers trigger a similar abnormality, is particularly interesting and may hold true for other, less well-studied TF genes. In addition to cooperative binding, TFs may recruit – or assist the binding of – other factors to chromatin. ‘Pioneer’ TFs, such as the HI-associated forkheadbox A1 and 2 (FOXA1/2) and SPI1 (also known as PU.1), bind ‘closed’ chromatin, making it accessible to non-pioneer TFs that act either alone or through interactions with chromatin remodelers (Barozzi et al., 2014; Cirillo et al., 2002; Heinz et al., 2010; Li et al., 2012; Zaret, 2018).

The functional impairment of protein complexes in disease has been recently summarised by Bergendahl et al., 2019, who discuss possible mechanisms by which disease-causing mutations alter protein structure. This, in turn, may result in aberrant complex formation by affecting the protein stoichiometry or by inhibiting other protein interactions, thus causing abnormal cell and tissue functions, ultimately leading to disease. Therefore, the number of TFs may influence the function of other TFs when transcriptional regulation depends on TF-cofactor complexes.

Single-molecule analysis of the stoichiometry of TF complexes can be performed in both living or fixed cells. The proximity of molecules, as an implicit measure of complex formation, can be analysed by Förster resonance energy transfer (FRET) (Dikovskaya et al., 2019; Szalóki et al., 2015) or bimolecular fluorescence complementation (BiFC) (Hu and Kerppola, 2003; Moustaqil et al., 2018; Papadopoulos et al., 2015) (see Table 2 and Fig. 2). However, steric hindrance and the need for correct three-dimensional orientation of TF complexes are main limiting factors of these methods, and may cause false-negative results. FRET, FCS and combined SPIM-fluorescence cross-correlation spectroscopy (SPIM-FCCS) have been used in combination to show that FOS forms homodimers that are also capable to bind chromatin when JUN is not present in equal concentrations to FOS (Szalóki et al., 2015). As the formation and transcriptional regulatory activity of FOS homodimers were suggested to play a role in oncogenesis, the stoichiometry of TF complexes and their activity have been investigated by combining different quantitative approaches in the context of *FOS* overexpression (Szalóki et al., 2015). TF complexes at specific nuclear compartments or gene loci, which are rendered visible in BiFC or undergo energy transfer in FRET, may also be imaged by using SRM (Kwon et al., 2017; Yamamoto et al., 2014) (see Table 2 and Fig. 2). Furthermore, one can analyse how the stoichiometry of TF complexes changes during development or varies across cells in the same tissue. This is possible by using FCS, as in studies regarding the homodimerization of Scr on chromatin (Papadopoulos et al., 2015; Papadopoulos et al., 2019; Rosales et al., 2013; Skouloudaki et al., 2019; Szalóki et al., 2015; Vukojevic et al., 2010) and *FOS* (Szalóki et al., 2015). FCS has also been applied to study the formation of heterocomplexes between MYC and the transcriptional regulator MAX (Rosales et al., 2013). Oligomerisation of the mitochondrial TF TFAM on chromatin was similarly investigated using fast-scanning stimulated emission depletion (STED) microscopy (Heller et al., 2013). Moreover, TF complexes have been studied by using dual labelling strategies. For example, Savatier and co-workers used two-photon FCCS to investigate the interactions between alpha and beta estrogen receptors (ESR1 and ESR2, respectively) or between ESRs and TIF2 (Savatier et al., 2010). The colocalisation of glucocorticoid receptor (NR3C1) and its interacting protein GRIP1, as well as of the transcriptional activators BMAL (aslo known as ARNTL) and CLOCK was similarly visualised by using dual-colour single-particle tracking (SPT) (Gebhardt et al., 2013). Thus, TF complexes can be quantitatively analysed in time and in space to provide molecular insights into how their interactions and concentrations affect entire regulatory networks. To uncover the roles of TFs in HI, their interactome needs to be thoroughly investigated and quantitative microscopy methods should be used to characterise the potentiially abnormal TF function based upon aberrant TF stoichiometries.

## Target site number, accessibility and binding

The cell- and tissue-specific sensitivity to TF levels also depend on the number of TF target-binding sites on chromatin and their accessibility – which might be subject to developmental control, as well as how TF molecules dynamically move in nuclei and undergo various interactions with chromatin, until they find and bind to their target sites. On average, the number of TF molecules per cell is between thousands and tens-of-thousands (Simicevic et al., 2013). This is roughly one order of magnitude higher than the number of specific TF binding sites, which is on average several hundreds to a few thousands (Ganapathi et al., 2011; Johnson et al., 2007; Palii et al., 2011; Robertson et al., 2007). Therefore, the number of binding sites of a given TF in a certain cell type cannot always explain why reduced TF amounts may not suffice for proper TF function. Additionally, genes controlled by a single TF may be more sensitive to molecular numbers than those controlled by two or more TFs, making the correlation between the ‘number of TF molecules’ and the ‘number of TF-binding sites’ far from straightforward. The complexity increases when we consider the existence of mechanisms, which overcome the dependence of transcriptional control on the stoichiometry of TF molecules to binding sites, i.e. by allowing the expression of important developmental genes to also depend on collaborating TFs. This is achieved by the evolutionary ‘addition’ of such ‘collaborating’ TF-binding sites into enhancers. For instance, the presence of both Zld- and Dorsal (Dl)-binding sites in an enhancer of the *short gastrulation* (*sog*) gene guarantees the potentiation of *sog* transcription in the early the *Drosophila* embryo, such that all cells express the same amount of *sog* – even in positions where the Dl morphogen TF concentration is low (Papadopoulos and Tomancak, 2019; Yamada et al., 2019). In this case, additional Zld-binding sites convert *sog* expression from ‘analog’ – i.e. proportional to Dl concentration along the Dl dorsoventral gradient – to ‘digital’, whereby Zld guarantees efficient *sog* expression as long as Zld-binding sites are present in the *sog* enhancer (Papadopoulos and Tomancak, 2019; Yamada et al., 2019). Thus, studies from the fly shed light onto how some target genes maintain robust expression, even upon limiting amounts of the TFs that regulate them. Such collaborating and ‘potentiating’ TFs may themselves be expressed in a tissue-specific manner. In this case, expression of the same target gene across different tissues or of different target genes in the same tissue both depend on the amount of TF. This might explain why certain TFs result in HIs in one tissue but not in another. Generally, the number of TF-binding sites may strongly influence the expression of specific genes in the same tissue or of the same gene in some tissues only and not in others, making gene expression less, or more, sensitive to TF numbers. Therefore, TF HIs that result in developmental abnormalities or disease frequently manifest in a subset of tissues, at distinct developmental stages or are due to the mis-regulation of only some of their target genes.

The binding affinity of TFs on their DNA-binding sites is another important factor required for normal gene regulation (Levine, 2010). This chiefly depends on the binding site sequence and defines how ‘strongly’ a TF binds to this site. Low-affinity binding sites are, thus, less likely to be bound when TF concentrations are low and, therefore, may require higher numbers of TFs and/or cofactors for transcriptional regulation (Arthur et al., 2017; Tsai et al., 2017). This may serve as an additional control mechanism for differential gene expression in space and time. In *Drosophila,* the Hox TF Ultrabithorax can bind low-affinity target sites on certain *shavenbaby* enhancers when complexed with the Hox cofactors Extradenticle and Homothorax. This confers region-specific control for the formation of epidermal denticle belts in the embryonic cuticle (Crocker et al., 2015; Tsai et al., 2017). While low-affinity binding sites render gene expression more sensitive to TF levels, their existence can be important for safeguarding the specificity of TF binding. This is of particular importance for the specification, development and differentiation of body structures. Additionally, it is an important mechanism to control and exploit the function of TFs that exhibit very similar binding behaviours, such as Hox TFs. As a result, the transcriptional output of genes with multiple low-affinity binding sites can be dosage dependent (Crocker et al., 2015; Driever and Nüsslein-Volhard, 1988; Giorgetti et al., 2010; Lorberbaum and Barolo, 2013; Ochoa-Espinosa et al., 2005; Ramos and Barolo, 2013; Stewart-Ornstein et al., 2013; Struhl et al., 1989; Tsai et al., 2017). This, in turn, enables cells to acquire distinct developmental fates based on their position along a TF concentration gradient (Driever and Nüsslein-Volhard, 1988; Lorberbaum and Barolo, 2013; Ochoa-Espinosa et al., 2005; Ramos and Barolo, 2013; Struhl et al., 1989). For instance, the homeodomain TF CUX1, a tumour suppressor, binds distal target enhancers in cultured human cells in concert with co-activators and cohesin. Loss of one *CUX1* allele abolishes CUX1 binding, and genes that contain multiple low-affinity CUX1-binding sites become mis-regulated (Arthur et al., 2017). Furthermore, Louphrasitthiphol et al. (2020) recently investigated the binding between microphthalmia-associated transcription factor (MITF) and chromatin, and suggested that low-affinity binding sites also act as a TF ‘reservoir’ in the genome. Chromatin immunoprecipitation sequencing (ChIP-seq) and single-molecule tracking (SMT) were used to show that the binding affinity between MITF and chromatin is increased by MITF acetylation, and that an acetylation-mutant MITF exhibits increased numbers of transient interactions with chromatin at low-affinity binding sites (Louphrasitthiphol et al., 2020). The authors suggested that low-affinity sites act as a reservoir for non-acetylated MITF, which is released upon acetylation in order to bind high-affinity sites (Louphrasitthiphol et al., 2020). Although both high- and low-affinity binding sites can favour TF specificity and facilitate differential gene expression, the number of TF-binding sites in enhancers, as well as their affinity, may also strongly affect the sensitivity of gene expression relative to TFs and, therefore, may result in TF HI.

Yet, cell- or tissue-specific transcriptional responsiveness of genes relative to the level of certain TFs may stem from the differential accessibility of their binding sites, and the set of TFs and cofactors targeting them. Both processes contribute to the kinetics of TF target site search and binding (Liu et al., 2014; Tsai et al., 2017). To find their specific binding sites, TFs ‘search’ chromatin by diffusion and transient, non-specific electrostatic interactions (Chen et al., 2014b; Elf et al., 2007; Liu et al., 2014; Louphrasitthiphol et al., 2020; Papadopoulos et al., 2010; Raccaud et al., 2019; Slutsky and Mirny, 2004; Voss et al., 2011; Vukojevic et al., 2010). Specific binding to their cognate sites depends on the strength of weak dipole interactions, such as hydrogen bonds and Van der Waals forces, which are exercised between amino acid (aa) residues of the TF and the DNA bases. Thus, the balance between non-specific and specific chromatin interactions determines the amount of TF molecules available to bind their cognate sites – an important determinant of how efficiently TFs find these specific binding sites.

The accessibility of binding sites depends on chromatin conformation and nuclear organisation, and may modulate the ability of TFs to efficiently search for and find their target sites. Chromatin organisation within cell nuclei is dynamic, cell type-specific and has been extensively studied with respect to its effect on gene expression. The degree of chromatin compaction affects TF binding and gene expression (Akhtar et al., 2013). Additionally, topologically associated domains (TADs), i.e. regions of self-interacting chromatin, are thought to regulate gene expression by establishing chromatin contacts within and between TADs, as well as by generating transcriptional microenvironments (Dixon et al., 2015; Tsai et al., 2017; reviewed by Gonzalez-Sandoval and Gasser, 2016). For example, Tsai and collaborators suggested that, in *Drosophila*, clustering of enhancers with low-affinity binding sites permits the formation of high local concentrations of the TF Ultrabithorax, which potentiates its binding interactions with chromatin (Tsai et al., 2017). TADs are also dynamic and cell type-specific, and have been studied with regard to differentiation (Kaur et al., 2013; Plachta et al., 2011; White et al., 2016), reprogramming (Beagan et al., 2016) and their role in *HOX* gene expression during limb development (Gebhardt et al., 2013; Langowski, 2017; Paakinaho et al., 2017; Rehó et al., 2020; Savatier et al., 2010; Vámosi et al., 2008). Nucleosome positioning further influences chromatin accessibility and, thereby, TF binding to gene regulatory elements (Chen et al., 2014a,b; Gebhardt et al., 2013; Hansen et al., 2017; Li et al., 2019; Mir et al., 2017; Normanno et al., 2015; Tirosh and Barkai, 2008; White et al., 2016; Wollman et al., 2019; Yao et al., 2006; Zhao et al., 2017). At the whole-genome level, histone modification signatures dictate chromatin accessibility and are subjected to developmental control (Bell et al., 2010). As such, the effect of binding site accessibility on TF-chromatin binding kinetics may trigger differential gene expression and differentiation biases between seemingly identical cells, even when the TFs mainly responsible for such processes are expressed in all cells at the same levels. In mouse preimplantation embryos, OCT4 exhibits differential chromatin-binding kinetics as early as at the 8-cell stage, which is one of the first determinants of differentiation bias towards inner cell mass or trophectoderm (Plachta et al., 2011). Moreover, the chromatin-binding kinetics of SOX2 and OCT4 can be used to predict cell fate (White et al., 2016). In pluripotent cells, OCT4 and SOX2 bind chromatin more stably than in extraembryonic cells (Kaur et al., 2013). Long-lived SOX2–chromatin binding is regulated by methylation of histone H3 at arginine 26 (H3R26), such that reduction in H3R26 methylation decreases the lifetime of SOX2-bound complexes. As a result, SOX2 target expression declines, and so do the numbers of pluripotent cells (White et al., 2016). These are excellent examples of how the kinetics of TF binding and the epigenetic landscape can influence development. Therefore, the dependence of gene expression on TF concentrations in a cell- and tissue-specific manner also relies on the number, affinity, accessibility and chromatin-binding kinetics of TF-binding sites.

It follows that quantitative analyses of TF-chromatin interaction kinetics and studies of cognate site configuration provide a deeper insight into gene- and cell-specific sensitivity to TF levels. FCS and SPT are used to obtain the fractions of freely diffusing TF molecules that seem to facilitate cognate site search, as well as of TF molecules bound to chromatin (see Fig. 2 and Table 2). For example, FCS studies of Scr in flies suggest that slowly diffusing TFs predominantly engage in rapid electrostatic interactions with chromatin, thereby reflecting the molecular movement TF molecules exhibit while searching for their specific DNA-binding sites (Papadopoulos et al., 2010; Vukojevic et al., 2010). Interestingly, the diffusion of MYC and P-TEFb – as measured by SPT – suggests that a binding site search may be TF- and target site-specific (Izeddin et al., 2014). By using SPT, enhancer clustering has been suggested to facilitate target-site search and binding (Liu et al., 2014). Chen and collaborators combined multi-focus SMT and FCS to study how mutant and wild-type SOX2 and OCT4 in mouse ESCs display differential binding dynamics on enhanceosomes, as well as the effects chromatin modifications have on TF molecules searching for their cognate DNA sites (Chen et al., 2014b). Once bound, the DNA-residence time of a TF reflects its binding affinity. SPT is better suited to measure long-lived – presumably specific – TF-DNA interactions. Monitoring of these interactions does not require very high temporal resolution and photobleaching can, thus, be avoided by time-lapse imaging experiments with longer ‘dark’ intervals between rounds of image acquisition. For example, by using reflected light-sheet microscopy (RLSM)-SPT, Gebhardt and colleagues compared the DNA-residence time of NR3C1 and of ESR monomers and dimers (Gebhardt et al., 2013). Mazza and collaborators combined SPT with FCS and FRAP to identify different proportions and residence times of mutant p53 (Mazza et al., 2012). FRAP can also be used to characterise TF-chromatin interactions by investigating their kinetic on- and off-chromatin rates, for instance, how TFs bind mitotic chromosomes. Here, the TFs exhibit high on-rates and reduced mobility during interphase, resulting in a more efficient search of TFs for binding sites (Raccaud et al., 2019). Furthermore, application of FRAP and SPT showed that SOX2-chromatin binding behaviour during mitosis is more dynamic than during interphase (Teves et al., 2016). FRAP experiments also identified that mutations in the high-mobility group domain of SOX2 largely abolished its clustering at the *POU5F1* enhancer, again suggesting that TFs can engage in different modes of chromatin-binding behaviour (Li et al., 2019). In addition, the chromatin-binding behaviour of TF heterocomplexes with other TFs has been studied by FCCS; particularly in cases where TF heterodimerisation is known to be required for DNA binding, such as for the dimers between FOS and JUN (Langowski, 2017), retinoic acid receptor (RAR) and retinoid X receptor (RXR) (Rehó et al., 2020), and ESR1/2 and TIF2 (Savatier et al., 2010). FRET has similarly been used to study chromatin binding of TF dimers and complexes, in some studies in combination with FCS or FCCS. Dimerisation and chromatin-bound intermediates of the basic helix-loop-helix leucine zipper (bHLH-LZ) domains of the TFs MYC and MAX were investigated by single-molecule FRET (smFRET) and FCS (Vancraenenbroeck and Hofmann, 2018). FCCS and FRET have also been used to study the chromatin-binding dynamics and inter-molecular distance between FOS and JUN in homo- and heterodimers (Szalóki et al., 2015; Vámosi et al., 2008). Additionally, ternary complexes between FOS, JUN and the NFκB subunit p65 have been investigated by BiFC-FRET (Shyu et al., 2008).

The signal-to-noise ratio in FCS and SPT can be increased by reducing the illumination volume, for example, by using lattice light-sheet microscopy (LLSM) in combination with SPT (LLSM-SPT) (Chen et al., 2014a; Mir et al., 2017), LLSM-FCS (Mir et al., 2017), RLSM-SPT (Gebhardt et al., 2013), highly inclined laminated optical sheet (HILO)-SPT (Hansen et al., 2017) or spatial light interference microscopy (SLIM)-SPT (Wollman et al., 2019), as well as by implementing sparse-excitation methodologies, such as 3D-STED (Li et al., 2019), single-particle tracking photo-activated localization microscopy (sptPALM) (Normanno et al., 2015), photo-activatable (PA)-FCS (White et al., 2016; Zhao et al., 2017) and multiphoton FCS (Yao et al., 2006) (see Table 2 and Fig. 2). The interaction of the GTPase RAP1 with nucleosomes and the resultant local opening of chromatin have also been studied *in vitro* by a combination of smFRET and TIRF (Mivelaz et al., 2020). Moreover, the spatial intranuclear variability in TF concentrations and chromatin-binding behaviour can simultaneously be addressed by novel methods, such as mpFCS (Krmpot et al., 2019; Papadopoulos et al., 2015) and 3D-SPT (Chen et al., 2014a). However, linking TF dynamics and concentration to their function by visualising specific genomic loci and their conformation has so far proven challenging.

To address these challenges, CRISPR/dCas9- and TALE-based labeling of specific DNA sequences can be used in live cells to visualise TF dynamics. However, the low signal-to-noise ratio has so far restricted this application to repetitive sequences (Chen et al., 2013; Knight et al., 2015; Ma et al., 2013; Thanisch et al., 2014). In a recent study, Li et al. targeted dCas9 to distal enhancers of the *Pou5f1* and *Nanog* genes, to link clusters of BRD4 and enhancers with active transcription (Li et al., 2020). However, the authors did not address the dynamic behaviour that might underlie such interactions. Specific conformations of TF-binding sites can be analysed using SRM in combination with chromatin markers associated with accessibility and active transcription (Cai et al., 2019). The kinetics and variability of TF-chromatin interactions can be analysed by quantitative microscopy at high temporal or spatial resolution. These methodologies allow to link the intercellular variability of TF-chromatin interactions and gene expression to gene- and cell-specific target site availability, and – from there – to further understand the role of TF-binding dynamics in the context of HIs.

## Stochastic gene expression

Although highly significant, TF-chromatin interactions alone do not sufficiently explain the significance of TF stoichiometry underlying HIs. Aberrant TF numbers can also affect gene expression because this in itself is an inherently stochastic process. Transcription is a ‘noisy’ process and, for a plethora of genes, it occurs in bursts, resulting in temporally fluctuating levels of mRNA (Bothma et al., 2014; Chubb et al., 2006; Fukaya et al., 2016; Larsson et al., 2019; Lee et al., 2019; Rodriguez et al., 2019). Because the frequency of transcriptional bursts depends on TF concentration (Senecal et al., 2014), when TF numbers are low, transcriptional noise can become a limiting factor for the normal expression of some genes and may result in abnormal function. The aforementioned stochasticity further results in temporally and spatially variable gene expression within a cell, and elicits heterogeneity between cells in both prokaryotic (Kierzek et al., 2001; Ozbudak et al., 2002; Wolf and Arkin, 2002) and eukaryotic organisms (Blake et al., 2003; Raser and O'Shea, 2004). In mammals, gene expression noise occurs both during development (Abranches et al., 2014; Mohammed et al., 2017; Olsson et al., 2016; Trapnell et al., 2014) and in disease (Avraham et al., 2015; Shaffer et al., 2017; Tirosh et al., 2016).

In bacteria, auto-activation of the competence TF ComK confers resistance to environmental stress by transcribing genes involved in the uptake of DNA (Maamar and Dubnau, 2005; Smits et al., 2005; Süel et al., 2006). Perturbing ComK levels by upregulating the rate of transcription and downregulating translation decreased the noise of ComK-dependent gene expression and reduced overall competency. This observation underlines that a subset of cells may exploit this cell-to-cell variability – introduced by transcriptional noise – to confer a group advantage (Maamar et al., 2007; Ozbudak et al., 2002; Süel et al., 2007; Thattai and van Oudenaarden, 2001). Albeit shown in a simple system, this indicates that stochasticity can result in transcriptional heterogeneity and increase the level of ‘fitness’ in a population. Similarly, in multicellular organisms, perturbations in TF numbers may amplify the cell-to-cell variability of transcriptional programmes. For example, in humans, deletions of one allele of the TF-encoding gene *NKX3.1* have been described in ∼40% of prostate cancers (Bova et al., 1993; Bowen et al., 2000; Macoska et al., 1995). Mouse models recapitulating this deletion display NKX3.1 HI, aberrant target gene expression and progressive prostate diseases (Abdulkadir et al., 2002; Magee et al., 2003). Transcriptional ‘bursting-like’ behaviour and the resulting cell-to-cell variability have been specifically associated with HIs of tumour suppressor genes (Kemkemer et al., 2002). The inactivation of a single allele of *NF1* results in neurofibromas in humans (Fahsold et al., 2000; Hoffmeyer et al., 1998). Furthermore, cultured melanocytes derived from patients with a single functional *NF1* allele exhibited variable dendritic outgrowth, owing to increased stochastic transcriptional noise compared to normal melanocytes (Kemkemer et al., 2002). Overall, such variabilities can lead to excessive divergence in gene expression, even among cells of the same lineage that feature very similar transcriptional programmes. Such paradigms underscore the complexity of TF HIs. That is, gene transcription might only become intolerably impaired in a subset of functionally identical cells – yet still lead to abnormal organ function and disease. Therefore, although it is established that low TF concentrations significantly influence ‘bursty’ gene expression, more studies will be needed to identify which target genes become the most severely impaired.

The inherently stochastic nature of transcription, discussed above, can be quantified by using single-molecule and single-cell methodologies and, thus, be linked to TF HIs. The dynamics of transcription can be observed *in vivo* by incorporating labelled nucleotides to monitor the production of nascent transcripts (Morisaki et al., 2014) or it can be visualised at specific loci by smFISH (Hsu et al., 2017; Kochan et al., 2015; Mehta et al., 2018; Titlow et al., 2018). Tagging the untranslated regions of endogenous genes with stem-loop-encoding sequences of bacteriophages MS2 or PP7, fused to a fluorescent reporter, allows the visualisation of nascent RNA and, thus, is suitable to track the expression of genes of interest (Bothma et al., 2014; Katz et al., 2018). Intercellular variability of transcription can be quantified by smFISH and scRNA-seq (Beach et al., 1999; Bertrand et al., 1998; Guo et al., 2017; Halpern et al., 2017; Hocine et al., 2013; Treutlein et al., 2016; Yan et al., 2013). Further, advances in scRNA-seq have allowed the analysis of allele-specific ‘bursting’ to be used as a means of identifying imbalances of gene expression (Borel et al., 2015; Chen et al., 2016; Deng et al., 2014; Faddah et al., 2013; Jiang et al., 2017; Kim and Marioni, 2013; Marks et al., 2015). In other studies, the cellular transcriptional activity has been inferred by examining localisation and dynamics of the transcriptional machinery (Cho et al., 2018; Cisse et al., 2013; Li et al., 2019; Steurer et al., 2018). From this, it becomes clear that to link TF behaviour to gene expression at high temporal and spatial resolution, studies often have to utilise both live- and fixed-cell methods to obtain complementary information. For example, the local concentration and dynamics of SOX2, CDK9, BRD4 and MED22 were measured by 3D STED and FRAP, and nascent *POU5F1* transcripts were visualised by MS2-MCP labelling (Li et al., 2019). This correlation between local concentrations of TFs and gene expression leads to the final TF HI-influencing mechanism explored in this Review: the formation of condensates.

## Control of TF function through the formation of condensates

In recent years, phase separation of proteins has gained substantial attention in biological research. The formation of biomolecular condensates has been implicated in a plethora of cellular functions. These are as diverse as membrane-less organelles, such as the nucleolus (Feric et al., 2016), as well as the normal and abnormal variants of widely studied proteins, such as FUS, G3BP1, TDP43 (officially known as TARDBP) and BRD4 (Han et al., 2020; McGurk et al., 2018; Niaki et al., 2020; Riback et al., 2020; Yang et al., 2020) which are implicated in disease pathogenesis. However, our understanding of the biological meaning of condensate formation, particularly by TFs, remains incomplete. As such, phase separation of TFs, aiding nuclear compartmentalisation and gene regulation, is a relatively novel concept (Boija et al., 2018).

Phase separation of proteins depends on physico-chemical conditions, such as protein concentration, charge, 3D structure and cellular pH (Taratuta et al., 1990; Wang et al., 2018). The thermodynamics of phase-separated systems predicts that protein concentration inside condensates is higher than in the surrounding dilute phase (Klosin et al., 2020). Condensates may possess liquid, gel or solid-like properties but the biological consequences of such phase-separated entities – and whether they are beneficial or not  – remain ill-defined (Brangwynne et al., 2009; Conicella et al., 2016; Kato et al., 2012; Lin et al., 2015; Strom et al., 2017). Condensates exhibit rapid protein exchange with the surrounding dilute phase, movement within the dense phase, as well as fusion and fission phenomena during their formation and maturation (Brangwynne et al., 2009; Handwerger et al., 2005; Phair and Misteli, 2000; Strasser et al., 2008). Although the exact biophysical mechanism of condensate formation remains elusive, weak multivalent interactions between intrinsically disordered regions seem to be the main driver (Dzuricky et al., 2020; Kato et al., 2012; Lin et al., 2017; Sabari et al., 2018; Wei et al., 2017).

As previously discussed, the intranuclear concentration of some TFs substantially influences gene regulation and function. Since condensate formation buffers concentration and functionally compartmentalises the nucleus (Klosin et al., 2020), condensates can control local TF concentrations. Super-enhancers are clusters of enhancers that accumulate components of the transcriptional machinery. They are thought to harness the formation of condensates to favour biochemical reactions through local increases in TF concentration and the formation of compartmentalised reaction/diffusion networks, without excluding additional mechanisms. In this way, the molecular crowding brings regulatory sequences and promoters into close proximity (Hnisz et al., 2013), and such reversible local reaction/diffusion networks favour gene expression. Such a process can depend on nucleation events triggered by physiological (e.g. NF-κB in inflammation; Nair et al., 2019), developmental (e.g. Prospero in neural differentiation; Liu et al., 2020) or molecular processes (e.g. depletion of proteins such as the Mediator complex and BRD4; Sabari et al., 2018). The high-density assemblies of transcriptional machinery components at enhancers is, by definition, substantially assisted by the formation of condensates. This is because TFs, e.g. SOX2, OCT4 and NANOG (Boija et al., 2018), co-activators, e.g. BRD4 and MED1 (Sabari et al., 2018) and RNAPolII (Boehning et al., 2018; Cho et al., 2018) all form condensates. However, to what extent this depends on condensate formation alone or on clustered DNA-binding interactions, remains under investigation (Li et al., 2020). So far, studies of the function of TF condensates in the regulation of gene expression remain scarce and have mostly focussed on linking condensate formation to transcriptional output (Li et al., 2020; Sabari et al., 2018), examining silencing of genes, such as *prospero* in *Drosophila* (Liu et al., 2020), and investigating diseases, such as HOXD13-associated synpolydactyly (Basu et al., 2020). Alternatively, condensate formation has been recently proposed to be involved in buffering the concentration of TFs in the dilute phase (Klosin et al., 2020), thereby dampening the variability of TF concentration. Although the universality of such functions remains to be experimentally confirmed, it is intriguing that many TFs depend on a stringent regulation of their local concentration for normal function. Examples include PAX6/SOX2 during ocular development (Matsushima et al., 2011), OCT4 in the preimplantation embryo (Gerovska and Araúzo-Bravo, 2019) and NANOG during blastocyst formation (Bessonnard et al., 2014). Therefore, formation of condensates and how they control TF variability across cells warrant further investigation in the context of HIs.

Understanding the biological function of TF condensate formation requires the characterisation of their constituents, their subcellular location and their dynamic behaviour over time. Fast scanning microscopy – such as confocal spinning-disk (CSD) microscopy and LLSM – is used to observe cellular localisation and co-partitioning of proteins into condensates (Chong et al., 2018; Sabari et al., 2018), and can be quantified by SRM (Cai et al., 2019; Chong et al., 2018; Sabari et al., 2018) (see Table 2 and Fig. 2).

The mobility of TFs within condensates, and the exchange of molecules between condensates and the environment, have been measured by FRAP, FCS and SPT. Condensates formed by transcriptional machinery components, such as BRD4 and MED1 (Boija et al., 2018; Cai et al., 2019; Chong et al., 2018; Gibson et al., 2019; Guillén-Boixet et al., 2020; Klosin et al., 2020; Liu et al., 2020; Sabari et al., 2018; Teves et al., 2016; Zamudio et al., 2019), and TF condensates, such as OCT4 (Boija et al., 2018), have been also studied by FRAP. Additionally, the formation of TF condensates driven by low complexity domains has been studied by FRAP and SPT (Chong et al., 2018). Stress-induced condensates, e.g. YAP, have also been studied by FRAP (Cai et al., 2019), and the formation and maturation of G3BP1-related stress granules have recently been investigated by FRAP and FCS (Guillén-Boixet et al., 2020).

It will be interesting to further investigate the biological functions of TF condensates through a combination of single-molecule approaches to link TF numbers and their dynamic behaviour to gene expression and HIs.

## Conclusions

Here, we have outlined how TF abundance and dynamic interactions with chromatin are required for normal development and how abnormalities in such regulation can result in disease.

We have discussed the key factors and cellular functions that control TF abundance in cells and tissues. TFs undergo complex kinetic interactions with chromatin; therefore, investigating TF numbers and molecular movement at the cellular and tissue levels is essential. Studied paradigms of dosage-sensitivity and HI emphasise how recent quantitative microscopy advances help researchers to investigate such complex TF interactions and their malfunction in disease. To date, such information on TFs and TF-cofactor complexes can be obtained with high spatiotemporal resolution. When these methodologies are applied to multiple cell types and disease models, they help to understand the molecular underpinnings of HI-associated diseases. However, a remaining challenge is to simultaneously study the behaviour of collaborating TFs and TF-complexes in live cells and tissues. This shortcoming is likely to result from the limitations of fluorescence microscopy to faithfully investigate several differently labelled proteins at the same time. Mass spectroscopy, RNA-seq and ChIP-seq methods do provide much broader, albeit static, datasets of TF binding and protein and mRNA abundances in cells but lack the dynamic information of live systems. A further challenge is to combine temporal and spatial super-resolution methods so that the mobility and chromatin-binding dynamics of individual TFs can be investigated at specific genomic loci. As discussed, this is currently limited to repetitive loci, which can be studied in tandem using the same fluorescent probe. Non-repetitive DNA sequences require a large number of sequence-specific probes to sufficiently increase the signal-to-noise ratio for fluorescence imaging (Chen et al., 2013; Knight et al., 2015; Ma et al., 2013; Thanisch et al., 2014).

Nevertheless, when studying the function of fluorescently labelled TFs, microscopy methodologies have become sensitive enough to detect molecules at low, physiologically relevant concentrations and by using low excitation power, which largely preserves the normal cell function. In fact, smaller and brighter fluorescent tags have been identified (Govindan et al., 2018; Oliinyk et al., 2019) but tagging genes non-disruptively does necessitate functional, i.e. genetic, validation of the endogenous behaviour of TFs. Additionally, the generation of further tissue and live animal models will explain the aberrant function(s) of TFs at the molecular level, in a biologically relevant context. For example, heterozygous LOF mutations of the widely expressed transcriptional co-activator YAP1 cause diverse defects in patients, such as ocular abnormalities or craniofacial/intellectual disabilities, suggesting that the observed phenotypes are the result of tissue-specific HIs of *YAP1* (Williamson et al., 2014). The generation of animal models to study the underlying mechanism of these defects will facilitate understanding the phenotypic variability observed in human patients by means of quantitative microscopy methodologies.

From a translational perspective, new animal models will enable the detection of early developmental transcriptional and signalling defects that normally lead to disease onset and progression. This will be possible by combining high spatial and temporal resolution microscopy – as discussed in this Review – with *in vitro* quantification of TF transcriptomes and interactomes. To date, most studies on TF function have addressed the impact of their mutations in disease only qualitatively. The information on intracellular TF concentrations and chromatin-binding behaviour is scarce, and may have been underappreciated. Until now, computational and bioinformatics analyses, coupled to machine learning and high-throughput screening, have been predominantly deployed to predict TF binding sites (Elmas et al., 2017; Shen et al., 2018) or TF-binding behaviour based on DNA structure (Zhou et al., 2015). Further approaches need to be developed to investigate the levels and molecular behaviour of TFs, and to predict transcriptional outcomes. To this end, the ample availability of current and future single-cell transcriptome datasets should be exploited to study how TF dosage affects gene transcription. This will enable us to understand the underlying mechanisms and the causal relationships between abnormal TF numbers, impaired TF behaviour and the manifestation of disease.
